# Crystal structure of di­chlorido­bis­(1,3-diazinane-2-thione-κ*S*)cadmium

**DOI:** 10.1107/S205698901502109X

**Published:** 2015-11-14

**Authors:** Ghazala Naz, Muhammad Nawaz Tahir, Saeed Ahmad, Anvarhusein A. Isab, Mohammed Fettouhi

**Affiliations:** a Department of Chemistry, University of Engineering and Technology, Lahore 54890, Pakistan; b Department of Physics, University of Sargodha, Sargodha, Pakistan; c Department of Chemistry, King Fahd University of Petroleum and Minerals, Dhahran 31261, Saudi Arabia

**Keywords:** crystal structure, cadmium chloride, 1,3-diazinane-2-thione

## Abstract

In the structure of the title compound, [CdCl_2_(C_4_H_8_N_2_S)_2_], the Cd^II^ atom is coordinated by two chloride ions and two 1,3-diazinane-2-thione (Diaz) mol­ecules through their S atoms. The geometry around the Cd^II^ atom is distorted tetra­hedral, with bond angles in the range 101.55 (7)–117.91 (8)°. The CH_2_ groups of one Diaz ligand are disordered over two sets of sites with an occupancy ratio of 0.711 (12):0.289 (12). The mol­ecular structure is stabilized by intra­molecular N—H⋯Cl hydrogen-bonding inter­actions, generating a butterfly *syn* conformation. Inter­molecular N—H⋯Cl and N—H⋯S inter­actions lead to the formation of a three-dimensional network structure. The structure has been determined from a crystal twinned by nonmerohedry, by a 180° rotation around the reciprocal *c* axis. The twin ratio refined to 0.8866 (6):0.1134 (6).

## Abstract 2

In di­chlorido­bis­(1,3-diazinane-2-thione-κ*S*)cadmium(II), the Cd^II^ atom is bound to two chloride anions and two thione ligands *via* their S atoms. The geometry around the Cd^II^ atom is distorted tetra­hedral, with the bond angles in the range 101.61 (3)–118.00 (3)°. Intra­molecular N—H⋯Cl hydrogen-bonding inter­actions stabilize a butterfly *syn* mol­ecular conformation.

## Chemical context   

Cadmium is considered to be a soft Lewis acid and possesses high affininty towards sulfur donor ligands such as thio­nes. Upon exposure to living systems, it preferablly inter­acts with sulfur-containing biomoleules. Therefore, complexes of cadmium with thio­nes are important as structural models to understand metal–sulfur inter­actions in biological systems (Akrivos, 2001 ▸; Bell *et al.*, 2004 ▸). In view of this, the crystal structures of several cadmium complexes of thio­nes, such as imidazolidine-2-thione (Imt) and 1,3-di­azinane-2-thione (Diaz), have been reported (Ahmad *et al.*, 2012 ▸; Al-Arfaj *et al.*, 1998 ▸; Bell *et al.*, 2004 ▸; Lobana *et al.*, 2008 ▸; Malik *et al.*, 2010 ▸; Mahmood *et al.*, 2012 ▸, 2015 ▸; Wazeer *et al.*, 2007 ▸). The complexity of structures of the *L*_2_Cd*X*_2_ type (where *L* is a thione and *X* is a halide or pseudohalide) ranges from mononuclear tetra­hedral complexes to polymeric octa­hedral species. We have reported recently the crystal structures of three cadmium complexes of Diaz, namely, [CdI_2_(Diaz)_2_], [Cd(CH_3_COO)_2_(Diaz)_2_] and [Cd(Diaz)_4_]SO_4_ (Ahmad *et al.*, 2012 ▸; Mahmood *et al.*, 2012 ▸, 2015 ▸). To learn more about the structural aspects of cadmium complexes, we report here the crystal structure of a cadmium chloride complex of 1,3-diazinane-2-thione, *i.e.* [CdCl_2_(Diaz)_2_], (I). The spectroscopic properties of the compound have been reported previously (Wazeer *et al.*, 2007 ▸).

## Structural commentary   

**Figure 1:**
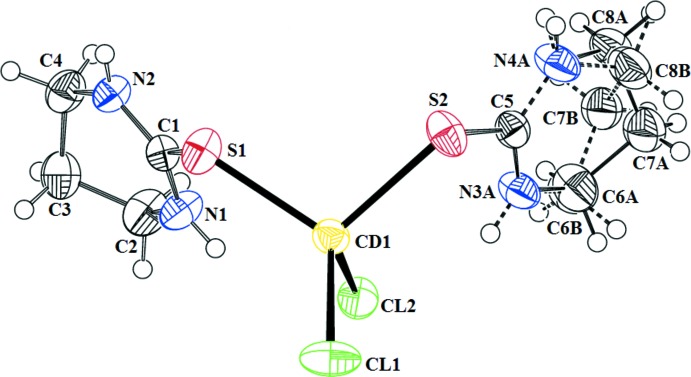
A view of the molecular structure of the title compound, showing the atom-numbering scheme. The displacement ellipsoids are drawn at the 50% probability level. The minor-occupancy C atoms are connected by dashed lines.

In the mol­ecular structure of (I) (Fig. 1 ▸), the Cd^II^ atom is bonded to two S atoms, each belonging to a Diaz mol­ecule, and two chloride ions. The coordination geometry at the Cd^II^ atom is distorted tetra­hedral, with the following bond angles: S—Cd—S = 105.08 (3)°, Cl—Cd—Cl = 101.61 (3)° and S—Cd—Cl in the range 108.91 (2)–118.00 (3)°. The Cl—Cd—Cl bond angle is significantly smaller than the other bond angles, which could be due to the involvement of Cl in intra­molecular (N—H⋯Cl) hydrogen bonding. The Cd—S and Cd—Cl bond lengths are in agreement with those reported for related compounds (Ahmad *et al.*, 2011 ▸, 2012 ▸; Al-Arfaj *et al.*, 1998 ▸; Bell *et al.*, 2004 ▸; Lobana *et al.*, 2008 ▸; Malik *et al.*, 2010 ▸; Mahmood *et al.*, 2012 ▸, 2015 ▸; Wazeer *et al.*, 2007 ▸). The two Diaz six-membered rings adopt half-chair conformations. In one of the two rings (the one involving atom S2), two of the methyl­ene C atoms (C7 and C8) are disordered over two positions. The SCN_2_ moieties of the Diaz ligands are essentially planar and the corresponding C—S and C—N bond lengths are in the ranges 1.730 (3)–1.731 (3) and 1.318 (3)–1.327 (3) Å, respectively. The C—S bond length is slightly longer than in the free ligand [1.720 (2) Å [Popovic *et al.*, 2001 ▸]. The shorter N—C(S) bond length compared to N—C(C) [1.456 (4) Å] is consistent with a significant N—C(S) double-bond character associated with an electronic delocalization towards the metal ion upon coordination. Compound (I) is related to that of the reported complexes [ZnCl_2_(Diaz)_2_] (Malik *et al.*, 2011 ▸) and [CdI_2_(Diaz)_2_] (Ahmad *et al.*, 2012 ▸) that both crystallize in space group *C*2/*c*. They show an equivalent degree of distortion from tetra­hedral configuration as in (I). However, in [CdCl_2_(Dmtu)_2_] and [CdBr_2_(Dmtu)_2_] (Dmtu = *N*,*N*′-di­methyl­thio­urea), the coordination geom­etry at the Cd^II^ atom is almost perfectly tetra­hedral (Ahmad *et al.*, 2011 ▸; Malik *et al.*, 2010 ▸).
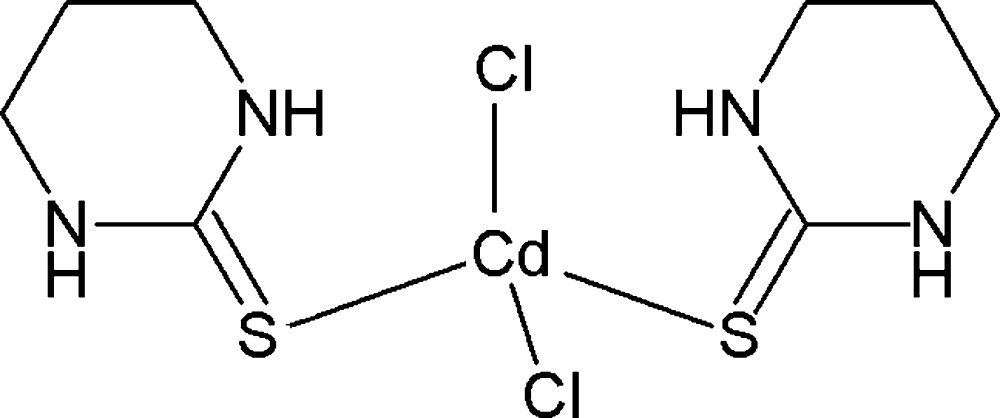


## Supra­molecular features   

**Figure 2:**
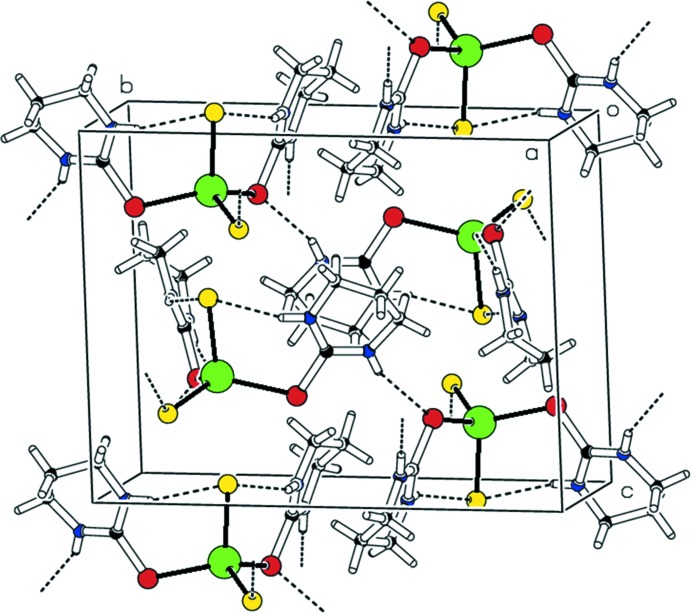
The packing diagram for (I), showing the hydrogen-bonding inter­actions. The minor-occupancy disordered atoms have been omitted for clarity.

Compound (I) shows both intra- and inter­molecular hydrogen-bonding inter­actions. One chloride anion (Cl2) is engaged in intra­molecular N—H⋯Cl hydrogen-bonding inter­actions with one N—H group of each of the two Diaz ligands (Table 1 ▸). This results in a butterfly *syn* conformation, where the two Diaz six-membered rings reside on the same side of the CdS_2_ plane. When such inter­actions are not effective, an *anti* conformation may be observed, where the two Diaz rings are located *anti* relative to the CdS_2_ plane. This situation is observed in [CdI_2_(Diaz)_2_] (Ahmad *et al.*, 2012 ▸). The second chloride (Cl1) anion undergoes inter­molecular hydrogen-bonding inter­actions with one N—H group of a Diaz ligand belonging to an adjacent complex mol­ecule, hence generating a chain structure along the *a* axis (Fig. 2 ▸). Furthermore, zigzag inter­chain N—H⋯S inter­actions take place, giving rise to a three-dimensional hydrogen-bonding network.

## Database survey   

A search of the Cambridge Structural Database (Groom & Allen, 2014 ▸) for cadmium complexes of 1,3-diazinane-2-thione yielded three structures including the above mentioned [CdI_2_(Diaz)_2_] (Ahmad *et al.*, 2012 ▸). Although the structure of [ZnCl_2_(Diaz)_2_] (Malik *et al.*, 2011 ▸) is similar to (I), the structure of a related mercury(II) complex is significantly different. It crystallizes in an ionic form, with {[Hg(Diaz)_2_]^2+^}_2_ cations and {[HgCl_4_]^2−^}_2_ anions (Popovic *et al.*, 2001 ▸).

## Synthesis and crystallization   

1,3-Diazinane-2-thione (Diaz) was prepared according to the literature procedure of Ahmad *et al.* (2012 ▸). The complex was prepared by adding a solution of Diaz (0.24 g, 2.0 mmol) in methanol (15 ml) to an aqueous solution (5 ml) of cadmium chloride (1.0 mmol, 0.21 g) and stirring the resulting mixture for 30 min. The colourless solution was filtered and the filtrate was kept at room temperature for crystallization. After 48 h, light-yellow crystals were obtained. The crystals were washed with methanol and dried in air (yield: 0.25 g, 0.60 mmol, 60%). The spectroscopic data of compound (I) have been reported previously (Wazeer *et al.*, 2007 ▸).

## Refinement details   

Crystal data, data collection and structure refinement details are summarized in Table 2 ▸. The crystal under investigation was found to be twinned by non-merohedry. The orientation matrices for the two components were identified using the program *CELL NOW* (Sheldrick, 2005 ▸), with the two components being related by a 180° rotation around the real/reciprocal axis [104]/(001). The two components were integrated using *SAINT* resulting in the following statistics: 7087 reflections (2024 unique) involved domain 1 only (mean *I*/σ = 23.7), 6799 reflections (1938 unique) involved domain 2 only (mean *I*/σ = 8.6) and 6567 reflections (1969 unique) the two domains (mean *I*/σ = 23.2).

The exact twin matrix identified by the integration program was found to be 1.00176 −0.00043 0.00606, −0.00069 −1.00042 0.00237, −0.52475 −0.00141 −1.00198. The structure was solved using direct methods with only the non-overlapping reflections of component 1. The structure was refined using the hklf 5 routine with all reflections of component 1 (including the overlapping ones) resulting in a BASF value of 0.1134 (6).

The C atoms of one Diaz moiety (C6/C7/C8) are disordered over two sets of sites, with an occupancy ratio of 0.715 (11):0.285 (11). Atoms N3*A* and N4*A* were constrained to have identical positions and displacement parameters as their equivalent partners in the major moiety, but their H atoms were included in the disorder model. Major and minor moieties were restrained to have similar geometries [SAME command in *SHELX2014* (Sheldrick, 2015 ▸)], and their atoms were subjected to a rigid-bond restraint (RIGU command in *SHELX2014*). The anisotropic displacement parameters of these C atoms were also subjected to a rigid-bond restraint (RIGU).

H atoms were placed at calculated positions and allowed to ride, with C—H and N—H distances of 0.97 and 0.86 Å, respectively, and with *U*_iso_(H) = 1.2*U*_eq_(C,N).

## Supplementary Material

Crystal structure: contains datablock(s) I, New_Global_Publ_Block. DOI: 10.1107/S205698901502109X/zl2641sup1.cif

Structure factors: contains datablock(s) I. DOI: 10.1107/S205698901502109X/zl2641Isup2.hkl

CCDC reference: 1435435

Additional supporting information:  crystallographic information; 3D view; checkCIF report

## Figures and Tables

**Table 1:** Hydrogen-bond geometry (Å, °)

*D*—H⋯*A*	*D*—H	H⋯*A*	*D*⋯*A*	*D*—H⋯*A*
N1—H1⋯Cl2	0.86	2.46	3.230 (3)	149
N2—H2⋯S2^i^	0.86	2.55	3.363 (2)	159
N3*A*—H3*C*⋯Cl2	0.86	2.46	3.196 (3)	144
N3*B*—H3*D*⋯Cl2	0.86	2.48	3.196 (3)	141
N4*A*—H4*C*⋯Cl1^ii^	0.86	2.44	3.270 (3)	162
N4*B*—H4*D*⋯Cl1^ii^	0.86	2.46	3.270 (3)	157

Symmetry codes: (i) 
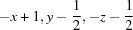
; (ii) 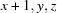.

**Table 2:** Experimental details

Crystal data	
Chemical formula	[CdCl_2_(C_4_H_8_N_2_S)_2_]
*M*_r_	415.67
Crystal system, space group	Monoclinic, *P*2_1_/*c*
Temperature (K)	296
*a*, *b*, *c* (Å)	8.5078 (8), 14.7201 (13), 12.0019 (10)
β (°)	101.016 (4)
*V* (Å^3^)	1475.4 (2)
*Z*	4
Radiation type	Mo *K*α
μ (mm^−1^)	2.11
Crystal size (mm)	0.40 × 0.30 × 0.28

Data collection	
Diffractometer	Bruker APEXII CCD
Absorption correction	Multi-scan (*TWINABS*; Sheldrick, 2009 ▸)
*T*_min_, *T*_max_	0.487, 0.589
No. of measured, independent and observed [*I* > 2σ(*I*)] reflections	3512, 3512, 3110
*R*_int_	0.037
(sin θ/λ)_max_ (Å^−1^)	0.657

Refinement	
*R*[*F* ^2^ > 2σ(*F* ^2^)], *wR*(*F* ^2^), *S*	0.029, 0.077, 1.05
No. of reflections	3512
No. of parameters	184
No. of restraints	50
H-atom treatment	H-atom parameters constrained
Δρ_max_, Δρ_min_ (e Å^−3^)	0.56, −1.18

Computer programs: *APEX2* and *SAINT* (Bruker, 2007 ▸) and *CELL NOW* (Sheldrick, 2005 ▸), *SHELXS97* (Sheldrick, 2008 ▸), *SHELXL2014* (Sheldrick, 2015 ▸) and *ORTEP-3 for Windows* (Farrugia, 2012 ▸).

## supplementary crystallographic information

### Crystal data

**Table:**

[CdCl_2_(C_4_H_8_N_2_S)_2_]	*F*(000) = 824
*M**_r_* = 415.67	*D*_x_ = 1.871 Mg m^−^^3^
Monoclinic, *P*2_1_/*c*	Mo *K*α radiation, λ = 0.71073 Å
*a* = 8.5078 (8) Å	Cell parameters from 3110 reflections
*b* = 14.7201 (13) Å	θ = 2.2–27.9°
*c* = 12.0019 (10) Å	µ = 2.11 mm^−^^1^
β = 101.016 (4)°	*T* = 296 K
*V* = 1475.4 (2) Å^3^	Block, light yellow
*Z* = 4	0.40 × 0.30 × 0.28 mm

### Data collection

**Table:**

Bruker APEXII CCD diffractometer	3512 independent reflections
Radiation source: fine-focus sealed tube	3110 reflections with *I* > 2σ(*I*)
Graphite monochromator	*R*_int_ = 0.037
Detector resolution: 7.6 pixels mm^-1^	θ_max_ = 27.9°, θ_min_ = 2.2°
φ and ω scans	*h* = −11→10
Absorption correction: multi-scan (TWINABS; Sheldrick, 2009)	*k* = 0→19
*T*_min_ = 0.487, *T*_max_ = 0.589	*l* = 0→15
3512 measured reflections	

### Refinement

**Table:**

Refinement on *F*^2^	Secondary atom site location: difference Fourier map
Least-squares matrix: full	Hydrogen site location: inferred from neighbouring sites
*R*[*F*^2^ > 2σ(*F*^2^)] = 0.029	H-atom parameters constrained
*wR*(*F*^2^) = 0.077	*w* = 1/[σ^2^(*F*_o_^2^) + (0.0414*P*)^2^ + 0.727*P*] where *P* = (*F*_o_^2^ + 2*F*_c_^2^)/3
*S* = 1.05	(Δ/σ)_max_ = 0.001
3512 reflections	Δρ_max_ = 0.56 e Å^−^^3^
184 parameters	Δρ_min_ = −1.18 e Å^−^^3^
50 restraints	Extinction correction: *SHELXL2014* (Sheldrick 2015), Fc^*^=kFc[1+0.001xFc^2^λ^3^/sin(2θ)]^-1/4^
Primary atom site location: structure-invariant direct methods	Extinction coefficient: 0.0020 (4)

### Special details

**Table:**

Geometry. All e.s.d.'s (except the e.s.d. in the dihedral angle between two l.s. planes) are estimated using the full covariance matrix. The cell e.s.d.'s are taken into account individually in the estimation of e.s.d.'s in distances, angles and torsion angles; correlations between e.s.d.'s in cell parameters are only used when they are defined by crystal symmetry. An approximate (isotropic) treatment of cell e.s.d.'s is used for estimating e.s.d.'s involving l.s. planes.
Refinement. Refinement of *F*^2^ against ALL reflections. The weighted *R*-factor *wR* and goodness of fit *S* are based on *F*^2^, conventional *R*-factors *R* are based on *F*, with *F* set to zero for negative *F*^2^. The threshold expression of *F*^2^ > σ(*F*^2^) is used only for calculating *R*-factors(gt) *etc*. and is not relevant to the choice of reflections for refinement. *R*-factors based on *F*^2^ are statistically about twice as large as those based on *F*, and *R*- factors based on ALL data will be even larger.

### Fractional atomic coordinates and isotropic or equivalent isotropic displacement parameters (Å^2^)

**Table:**

	*x*	*y*	*z*	*U*_iso_*/*U*_eq_	Occ. (<1)
Cd1	0.24350 (2)	0.25053 (2)	−0.17323 (2)	0.03632 (9)	
Cl1	0.00058 (10)	0.33253 (7)	−0.25626 (7)	0.0599 (2)	
Cl2	0.24104 (10)	0.26375 (5)	0.03652 (7)	0.04534 (18)	
S1	0.26254 (11)	0.08460 (5)	−0.22331 (6)	0.04513 (19)	
S2	0.50720 (10)	0.32086 (6)	−0.20300 (6)	0.0462 (2)	
N1	0.2258 (3)	0.04740 (16)	−0.0092 (2)	0.0449 (6)	
H1	0.1902	0.1020	−0.0077	0.054*	
N2	0.3317 (3)	−0.06371 (16)	−0.1046 (2)	0.0388 (5)	
H2	0.3724	−0.0789	−0.1622	0.047*	
C1	0.2737 (3)	0.01920 (18)	−0.1023 (2)	0.0330 (5)	
C2	0.2306 (5)	−0.0096 (2)	0.0905 (2)	0.0524 (8)	
H2A	0.1458	0.0081	0.1298	0.063*	
H2B	0.3322	−0.0017	0.1422	0.063*	
C3	0.2098 (4)	−0.1080 (2)	0.0549 (3)	0.0486 (8)	
H3A	0.2239	−0.1466	0.1215	0.058*	
H3B	0.1028	−0.1176	0.0113	0.058*	
C4	0.3314 (4)	−0.1317 (2)	−0.0156 (3)	0.0458 (7)	
H4A	0.4368	−0.1347	0.0324	0.055*	
H4B	0.3070	−0.1909	−0.0500	0.055*	
C5	0.6195 (3)	0.35738 (18)	−0.0751 (2)	0.0355 (6)	
N3A	0.5543 (3)	0.37659 (18)	0.0138 (2)	0.0446 (6)	0.715 (11)
H3C	0.4526	0.3700	0.0077	0.053*	0.715 (11)
C6A	0.6473 (10)	0.4084 (17)	0.1216 (8)	0.058 (4)	0.715 (11)
H6A	0.6716	0.3575	0.1733	0.069*	0.715 (11)
H6B	0.5846	0.4519	0.1553	0.069*	0.715 (11)
C7A	0.7995 (6)	0.4519 (5)	0.1047 (5)	0.0599 (18)	0.715 (11)
H7A	0.8663	0.4651	0.1778	0.072*	0.715 (11)
H7B	0.7759	0.5086	0.0639	0.072*	0.715 (11)
C8A	0.8876 (8)	0.3891 (5)	0.0382 (5)	0.0501 (17)	0.715 (11)
H8A	0.9821	0.4188	0.0214	0.060*	0.715 (11)
H8B	0.9206	0.3344	0.0815	0.060*	0.715 (11)
N4A	0.7753 (3)	0.3665 (2)	−0.0678 (2)	0.0503 (7)	0.715 (11)
H4C	0.8138	0.3587	−0.1284	0.060*	0.715 (11)
N3B	0.5543 (3)	0.37659 (18)	0.0138 (2)	0.0446 (6)	0.285 (11)
H3D	0.4519	0.3718	0.0043	0.053*	0.285 (11)
C6B	0.637 (2)	0.405 (4)	0.126 (2)	0.054 (9)	0.285 (11)
H6C	0.5985	0.3701	0.1837	0.065*	0.285 (11)
H6D	0.6163	0.4686	0.1374	0.065*	0.285 (11)
C7B	0.8121 (14)	0.3903 (11)	0.1362 (9)	0.051 (4)	0.285 (11)
H7C	0.8369	0.3264	0.1489	0.062*	0.285 (11)
H7D	0.8698	0.4242	0.2005	0.062*	0.285 (11)
C8B	0.864 (2)	0.4215 (12)	0.0283 (11)	0.055 (5)	0.285 (11)
H8C	0.8404	0.4855	0.0154	0.066*	0.285 (11)
H8D	0.9787	0.4129	0.0348	0.066*	0.285 (11)
N4B	0.7753 (3)	0.3665 (2)	−0.0678 (2)	0.0503 (7)	0.285 (11)
H4D	0.8239	0.3416	−0.1166	0.060*	0.285 (11)

### Atomic displacement parameters (Å^2^)

**Table:**

	*U*^11^	*U*^22^	*U*^33^	*U*^12^	*U*^13^	*U*^23^
Cd1	0.03251 (13)	0.03676 (13)	0.03966 (13)	0.00136 (8)	0.00685 (9)	0.00459 (8)
Cl1	0.0476 (4)	0.0886 (6)	0.0445 (4)	0.0300 (4)	0.0115 (3)	0.0120 (4)
Cl2	0.0537 (5)	0.0469 (4)	0.0391 (3)	−0.0049 (3)	0.0180 (3)	−0.0017 (3)
S1	0.0669 (5)	0.0369 (4)	0.0326 (3)	−0.0009 (3)	0.0120 (3)	0.0021 (3)
S2	0.0460 (4)	0.0624 (5)	0.0318 (3)	−0.0169 (4)	0.0118 (3)	0.0020 (3)
N1	0.0633 (17)	0.0344 (12)	0.0418 (12)	0.0098 (12)	0.0219 (12)	0.0036 (10)
N2	0.0451 (14)	0.0345 (12)	0.0377 (11)	0.0040 (10)	0.0103 (10)	−0.0032 (10)
C1	0.0331 (14)	0.0331 (13)	0.0325 (11)	−0.0029 (11)	0.0055 (10)	−0.0001 (10)
C2	0.076 (2)	0.0485 (18)	0.0372 (14)	0.0022 (16)	0.0223 (15)	0.0047 (13)
C3	0.059 (2)	0.0433 (17)	0.0426 (15)	−0.0060 (15)	0.0084 (14)	0.0090 (13)
C4	0.0532 (19)	0.0357 (15)	0.0456 (15)	0.0055 (14)	0.0024 (14)	0.0042 (12)
C5	0.0371 (15)	0.0351 (14)	0.0354 (12)	−0.0034 (11)	0.0093 (11)	0.0050 (10)
N3A	0.0331 (13)	0.0609 (16)	0.0413 (12)	−0.0057 (11)	0.0113 (10)	−0.0080 (11)
C6A	0.055 (4)	0.078 (9)	0.042 (3)	−0.014 (4)	0.011 (3)	−0.015 (4)
C7A	0.049 (3)	0.066 (4)	0.064 (3)	−0.009 (3)	0.010 (2)	−0.021 (3)
C8A	0.032 (3)	0.057 (4)	0.059 (3)	0.000 (3)	0.004 (2)	−0.006 (3)
N4A	0.0349 (14)	0.0725 (19)	0.0463 (13)	−0.0056 (12)	0.0151 (11)	−0.0070 (12)
N3B	0.0331 (13)	0.0609 (16)	0.0413 (12)	−0.0057 (11)	0.0113 (10)	−0.0080 (11)
C6B	0.042 (7)	0.07 (2)	0.046 (8)	−0.008 (7)	0.008 (5)	−0.015 (9)
C7B	0.044 (6)	0.058 (9)	0.050 (5)	−0.005 (5)	0.005 (4)	−0.015 (5)
C8B	0.036 (7)	0.067 (10)	0.060 (6)	−0.007 (7)	0.004 (5)	−0.008 (6)
N4B	0.0349 (14)	0.0725 (19)	0.0463 (13)	−0.0056 (12)	0.0151 (11)	−0.0070 (12)

### Geometric parameters (Å, º)

**Table:**

Cd1—Cl1	2.4361 (8)	N3A—C6A	1.458 (7)
Cd1—S1	2.5280 (8)	N3A—H3C	0.8600
Cd1—Cl2	2.5290 (8)	C6A—C7A	1.493 (12)
Cd1—S2	2.5571 (8)	C6A—H6A	0.9700
S1—C1	1.730 (3)	C6A—H6B	0.9700
S2—C5	1.731 (3)	C7A—C8A	1.510 (8)
N1—C1	1.327 (3)	C7A—H7A	0.9700
N1—C2	1.456 (4)	C7A—H7B	0.9700
N1—H1	0.8600	C8A—N4A	1.476 (6)
N2—C1	1.319 (3)	C8A—H8A	0.9700
N2—C4	1.464 (4)	C8A—H8B	0.9700
N2—H2	0.8600	N4A—H4C	0.8600
C2—C3	1.511 (5)	N3B—C6B	1.456 (15)
C2—H2A	0.9700	N3B—H3D	0.8600
C2—H2B	0.9700	C6B—C7B	1.484 (19)
C3—C4	1.497 (4)	C6B—H6C	0.9700
C3—H3A	0.9700	C6B—H6D	0.9700
C3—H3B	0.9700	C7B—C8B	1.518 (15)
C4—H4A	0.9700	C7B—H7C	0.9700
C4—H4B	0.9700	C7B—H7D	0.9700
C5—N4B	1.318 (4)	C8B—N4B	1.492 (13)
C5—N4A	1.318 (4)	C8B—H8C	0.9700
C5—N3B	1.324 (3)	C8B—H8D	0.9700
C5—N3A	1.324 (3)	N4B—H4D	0.8600
			
Cl1—Cd1—S1	118.00 (3)	N3A—C6A—H6A	109.5
Cl1—Cd1—Cl2	101.61 (3)	C7A—C6A—H6A	109.5
S1—Cd1—Cl2	108.91 (2)	N3A—C6A—H6B	109.5
Cl1—Cd1—S2	116.15 (3)	C7A—C6A—H6B	109.5
S1—Cd1—S2	105.08 (3)	H6A—C6A—H6B	108.1
Cl2—Cd1—S2	106.39 (3)	C6A—C7A—C8A	109.9 (8)
C1—S1—Cd1	109.50 (9)	C6A—C7A—H7A	109.7
C5—S2—Cd1	110.63 (9)	C8A—C7A—H7A	109.7
C1—N1—C2	123.1 (2)	C6A—C7A—H7B	109.7
C1—N1—H1	118.4	C8A—C7A—H7B	109.7
C2—N1—H1	118.4	H7A—C7A—H7B	108.2
C1—N2—C4	124.4 (2)	N4A—C8A—C7A	106.8 (5)
C1—N2—H2	117.8	N4A—C8A—H8A	110.4
C4—N2—H2	117.8	C7A—C8A—H8A	110.4
N2—C1—N1	118.9 (2)	N4A—C8A—H8B	110.4
N2—C1—S1	117.3 (2)	C7A—C8A—H8B	110.4
N1—C1—S1	123.8 (2)	H8A—C8A—H8B	108.6
N1—C2—C3	109.7 (2)	C5—N4A—C8A	123.9 (3)
N1—C2—H2A	109.7	C5—N4A—H4C	118.1
C3—C2—H2A	109.7	C8A—N4A—H4C	118.1
N1—C2—H2B	109.7	C5—N3B—C6B	127.0 (7)
C3—C2—H2B	109.7	C5—N3B—H3D	116.5
H2A—C2—H2B	108.2	C6B—N3B—H3D	116.5
C4—C3—C2	109.1 (3)	N3B—C6B—C7B	109.7 (14)
C4—C3—H3A	109.9	N3B—C6B—H6C	109.7
C2—C3—H3A	109.9	C7B—C6B—H6C	109.7
C4—C3—H3B	109.9	N3B—C6B—H6D	109.7
C2—C3—H3B	109.9	C7B—C6B—H6D	109.7
H3A—C3—H3B	108.3	H6C—C6B—H6D	108.2
N2—C4—C3	110.5 (2)	C6B—C7B—C8B	109.7 (18)
N2—C4—H4A	109.5	C6B—C7B—H7C	109.7
C3—C4—H4A	109.5	C8B—C7B—H7C	109.7
N2—C4—H4B	109.5	C6B—C7B—H7D	109.7
C3—C4—H4B	109.5	C8B—C7B—H7D	109.7
H4A—C4—H4B	108.1	H7C—C7B—H7D	108.2
N4B—C5—N3B	119.7 (3)	N4B—C8B—C7B	107.9 (11)
N4A—C5—N3A	119.7 (3)	N4B—C8B—H8C	110.1
N4B—C5—S2	117.9 (2)	C7B—C8B—H8C	110.1
N4A—C5—S2	117.9 (2)	N4B—C8B—H8D	110.1
N3B—C5—S2	122.4 (2)	C7B—C8B—H8D	110.1
N3A—C5—S2	122.4 (2)	H8C—C8B—H8D	108.4
C5—N3A—C6A	122.9 (4)	C5—N4B—C8B	117.5 (7)
C5—N3A—H3C	118.5	C5—N4B—H4D	121.3
C6A—N3A—H3C	118.5	C8B—N4B—H4D	121.3
N3A—C6A—C7A	110.8 (7)		
			
C4—N2—C1—N1	7.1 (4)	S2—C5—N3A—C6A	−179.3 (12)
C4—N2—C1—S1	−172.2 (2)	C5—N3A—C6A—C7A	24 (2)
C2—N1—C1—N2	−1.5 (4)	N3A—C6A—C7A—C8A	−51.6 (17)
C2—N1—C1—S1	177.7 (3)	C6A—C7A—C8A—N4A	55.3 (10)
Cd1—S1—C1—N2	−160.02 (19)	N3A—C5—N4A—C8A	5.9 (6)
Cd1—S1—C1—N1	20.8 (3)	S2—C5—N4A—C8A	−174.4 (4)
C1—N1—C2—C3	−29.8 (4)	C7A—C8A—N4A—C5	−34.2 (8)
N1—C2—C3—C4	53.7 (4)	N4B—C5—N3B—C6B	−2 (3)
C1—N2—C4—C3	19.8 (4)	S2—C5—N3B—C6B	178 (3)
C2—C3—C4—N2	−49.1 (3)	C5—N3B—C6B—C7B	−12 (5)
Cd1—S2—C5—N4B	157.6 (2)	N3B—C6B—C7B—C8B	43 (4)
Cd1—S2—C5—N4A	157.6 (2)	C6B—C7B—C8B—N4B	−61 (3)
Cd1—S2—C5—N3B	−22.8 (3)	N3B—C5—N4B—C8B	−16.5 (9)
Cd1—S2—C5—N3A	−22.8 (3)	S2—C5—N4B—C8B	163.2 (8)
N4A—C5—N3A—C6A	0.4 (12)	C7B—C8B—N4B—C5	47.6 (16)

### Hydrogen-bond geometry (Å, º)

**Table:**

*D*—H···*A*	*D*—H	H···*A*	*D*···*A*	*D*—H···*A*
N1—H1···Cl2	0.86	2.46	3.230 (3)	149
N2—H2···S2^i^	0.86	2.55	3.363 (2)	159
N3*A*—H3*C*···Cl2	0.86	2.46	3.196 (3)	144
N3*B*—H3*D*···Cl2	0.86	2.48	3.196 (3)	141
N4*A*—H4*C*···Cl1^ii^	0.86	2.44	3.270 (3)	162
N4*B*—H4*D*···Cl1^ii^	0.86	2.46	3.270 (3)	157
C7*B*—H7*C*···Cl1^iii^	0.97	2.85	3.769 (16)	159

Symmetry codes: (i) −*x*+1, *y*−1/2, −*z*−1/2; (ii) *x*+1, *y*, *z*; (iii) *x*+1, −*y*+1/2, *z*+1/2.

## References

[bb1] Ahmad, S., Altaf, M., Stoeckli-Evans, H., Isab, A. A., Malik, M. R., Ali, S. & Shuja, S. (2011). *J. Chem. Crystallogr.* **41**, 1099–1104.

[bb2] Ahmad, S., Amir, Q., Naz, G., Fettouhi, M., Isab, A. A., Rüffer, T. & Lang, H. (2012). *J. Chem. Crystallogr.* **42**, 615–620.

[bb3] Akrivos, P. D. (2001). *Coord. Chem. Rev.* **213**, 181–210.

[bb4] Al-Arfaj, A. R., Reibenspies, J. H., Isab, A. A. & Hussain, M. S. (1998). *Acta Cryst.* C**54**, 51–53.

[bb5] Bell, N. A., Clegg, W., Coles, S. J., Constable, C. P., Harrington, R. W., Hursthouse, M. B., Light, M. E., Raper, E. S., Sammon, C. & Walker, M. R. (2004). *Inorg. Chim. Acta*, **357**, 2091–2099.

[bb6] Bruker (2007). *APEX2* and *SAINT*. Bruker AXS Inc., Madison, Wisconsin, USA.

[bb7] Farrugia, L. J. (2012). *J. Appl. Cryst.* **45**, 849–854.

[bb8] Groom, C. R. & Allen, F. H. (2014). *Angew. Chem. Int. Ed.* **53**, 662–671. 10.1002/anie.201306438 24382699

[bb9] Lobana, T. S., Sharma, R., Sharma, R., Sultana, R. & Butcher, R. J. (2008). *Z. Anorg. Allg. Chem.* **634**, 718–723.

[bb10] Mahmood, R., Ghulam Hussain, S., Fettouhi, M., Isab, A. A. & Ahmad, S. (2012). *Acta Cryst.* E**68**, m1352–m1353. 10.1107/S1600536812041852 PMC3515111 23284338

[bb11] Mahmood, R., Hussain, S. G., Isab, A. A., Fettouhi, M., Fazal, A. & Ahmad, S. (2015). *J. Struct. Chem.* **56**, 463–467.

[bb12] Malik, M. R., Ali, S., Fettouhi, M., Isab, A. A. & Ahmad, S. (2010). *J. Struct. Chem.* **51**, 976–979.

[bb13] Malik, M. R., Vasylyeva, V., Merz, K., Metzler-Nolte, N., Saleem, M., Ali, S., Isab, A. A., Munawar, K. S. & Ahmad, S. (2011). *Inorg. Chim. Acta*, **376**, 207–211.

[bb14] Popovic, Z., Matkovic-Calogovic, D., Pavlovic, G., Soldin, Z., Giester, G., Rajic, M. & Vikic-Topic, D. (2001). *Croat. Chem. Acta*, **74**, 359–380.

[bb15] Sheldrick, G. M. (2005). *CELL NOW.* University of Göttingen, Germany.

[bb16] Sheldrick, G. M. (2008). *Acta Cryst.* A**64**, 112–122. 10.1107/S0108767307043930 18156677

[bb17] Sheldrick, G. M. (2009). *TWINABS*. University of Göttingen, Germany.

[bb18] Sheldrick, G. M. (2015). *Acta Cryst.* C**71**, 3–8.

[bb19] Wazeer, M. I. M., Isab, A. A. & Fettouhi, M. (2007). *Polyhedron*, **26**, 1725–1730.

